# Generation of Insulin-Producing Cells from the Mouse Liver Using β Cell-Related Gene Transfer Including *Mafa* and *Mafb*

**DOI:** 10.1371/journal.pone.0113022

**Published:** 2014-11-14

**Authors:** Haruka Nagasaki, Tokio Katsumata, Hisashi Oishi, Pei-Han Tai, Yukari Sekiguchi, Ryusuke Koshida, Yunshin Jung, Takashi Kudo, Satoru Takahashi

**Affiliations:** 1 Department of Anatomy and Embryology, Faculty of Medicine, University of Tsukuba, Tsukuba, Ibaraki, Japan; 2 Life Science Center, Tsukuba Advanced Research Alliance (TARA), University of Tsukuba, Tsukuba, Ibaraki, Japan; 3 International Institute for Integrative Sleep Medicine (WPI-IIIS), University of Tsukuba, Tsukuba, Ibaraki, Japan; Broad Institute of Harvard and MIT, United States of America

## Abstract

Recent studies on the large Maf transcription factors have shown that *Mafb* and *Mafa* have respective and distinctive roles in β-cell development and maturation. However, whether this difference in roles is due to the timing of the gene expression (roughly, expression of *Mafb* before birth and of *Mafa* after birth) or to the specific function of each gene is unclear. Our aim was to examine the functional differences between these genes that are closely related to β cells by using an *in vivo* model of β-like cell generation. We monitored insulin gene transcription by measuring bioluminescence emitted from the liver of insulin promoter-luciferase transgenic (MIP-Luc-VU) mice. Adenoviral gene transfers of *Pdx1/Neurod/Mafa* (PDA) and *Pdx1/Neurod/Mafb* (PDB) combinations generated intense luminescence from the liver that lasted for more than 1 week and peaked at 3 days after transduction. The peak signal intensities of PDA and PDB were comparable. However, PDA but not PDB transfer resulted in significant bioluminescence on day 10, suggesting that *Mafa* has a more sustainable role in insulin gene activation than does *Mafb*. Both PDA and PDB transfers ameliorated the glucose levels in a streptozotocin (STZ)-induced diabetic model for up to 21 days and 7 days, respectively. Furthermore, PDA transfer induced several gene expressions necessary for glucose sensing and insulin secretion in the liver on day 9. However, a glucose tolerance test and liver perfusion experiment did not show glucose-stimulated insulin secretion from intrahepatic β-like cells. These results demonstrate that bioluminescence imaging in MIP-Luc-VU mice provides a noninvasive means of detecting β-like cells in the liver. They also show that *Mafa* has a markedly intense and sustained role in β-like cell production in comparison with *Mafb*.

## Introduction

The development of pancreatic β cells is a highly orchestrated process that involves many extracellular stimulants and intracellular signaling pathways. At embryonic day 9.5 (E9.5), the pancreas evolves from the dorsal and ventral buds of the foregut's endodermal epithelium [1]. After gut rotation and fusion of the dorsal and ventral rudiments at around E11.5, the pancreatic epithelium initiates branching by sending finger-like epithelial protrusions into the surrounding mesenchyme [2]. As a branching duct-like structure forms, endocrine progenitors expressing neurogenin 3 within the trunks delaminate from the developing epithelium. Subsequently, progenitor cells successively differentiate into each hormone-producing cell under the hierarchical control of a lineage-specific molecular cascade [2]. Among the 5 different types of endocrine cells in the Langerhans islets, the development of β cells has been the most intensively investigated in relation to future complementation therapy against diabetes.

The Maf family of transcription factors, which is a member of the basic leucine-zipper (bZip) transcription factor family, are homologs of v-Maf, the oncogenic component of the avian retrovirus AS42 originally identified in the genome of chicken musculoaponeurotic fibrosarcoma (MAF), and are subdivided into 2 groups, small and large MAF proteins, according to their structure, function, and molecular size [3]–[5]. Three small MAF proteins, MAFF, MAFG, and MAFK, lack a transactivation domain and regulate genes important in hematopoiesis and the stress response by forming a heterodimer with other bZIP factors such as cap'n'collar (CNC) family proteins. To date, abnormalities in pancreatic development and glucose homeostasis of small maf knockout mice have not been reported [6]. In contrast, the large MAF proteins comprise 4 distinct proteins, MAFA, MAFB, c-MAF, and NRL, which are characterized by the presence of an N-terminal transactivation domain. In β-cell development, at E15.5, 50%, and 90% of insulin-positive cells express *Mafa* and *Mafb*, respectively; however, in adult mice, *Mafa* and *Mafb* are preferentially expressed in β cells and α cells, respectively [7]. Therefore, this switch from *Mafb* to *Mafa* expression in β cells probably has a crucial meaning for functional maturation in both mouse models and in human embryonic stem cell differentiation [7], [8].

Bioluminescence imaging (BLI) in small animals is a versatile and cost-effective technology that has high sensitivity and efficiency [9]. In β-cell research, BLI has been employed for the quantification of β-cell mass, monitoring of islet graft survival after transplantation, and detection of reporter gene expression [10]–[12]. Recently, we have shown that BLI of luciferase reporter mice, which can express luciferase under the control of the *Ins1* gene, could also be useful for the detection of fetal β-cell genesis in utero and for intrahepatic insulin gene activity [13], [14].

In this study, we aimed to examine the functional differences between *Mafa* and *Mafb* using an *in vivo* model of β-like cell conversion. Although these closely related genes each have their distinctive role in β-cell development (*Mafb*) and maturation (*Mafa*), whether the difference is due to the timing of the gene expression or to the characteristic features of each gene is unclear. Even though both *Mafa* and *Mafb* similarly activate insulin gene expression through the Maf recognition element (MARE) *in vitro*, the less-conserved N-terminal activation domain of the large Maf transcription factors raises the possibility of different activation potentials and/or downstream targets [7]. Here, we transduced β cell-related genes, including *Mafa* and *Mafb*, into the livers of insulin reporter mice and compared the transcriptional activities of insulin and glucose metabolism. Gene transfer including of *Mafb* could activate the insulin gene in the mouse liver. However, a combination gene transfer including *Mafa* demonstrated more efficient and sustainable insulin induction for most experiments when compared with *Mafb*. These findings provide insight into the reprogramming cell potency of the *Mafa* gene and the gene-specific functions of *Mafa* and *Mafb* in normal β-cell generation.

## Materials and Methods

### Animals

All experiments were performed in compliance with the relevant Japanese and institutional laws and guidelines and approved by the University of Tsukuba animal ethics committee (authorization number 13–072). Transgenic mice expressing luciferase under the control of the mouse *ins1* promoter [FVB/N-Tg(*Ins1-luc*)VUPwrs/J; Stock number: 007800; MIP-Luc-VU] were purchased from the Jackson Laboratory (Bar Harbor, ME, USA). Jcl:ICR mice purchased from Clea (Clea Japan, Tokyo, Japan) were mated for the experiments. Mice were euthanized at the appropriate time points with carbon dioxide gas.

### Preparation of recombinant adenovirus vectors

Recombinant adenoviruses expressing mouse *Pdx1*, *Neurod* (kindly gifted by Dr. S. Yoshida), *Mafa*, and *Mafb* were prepared using the ViraPower Adenoviral Gateway Expression kit (Life Technologies, Carlsbad, CA, USA). Each cDNA fragment was cloned into a pENTR4 entry vector. To create expression clones and produce recombinant adenoviruses, the pENTR4 inserts were transferred into pAd/CMV/V5-DEST destination vectors using the LR recombination reaction, and PacI-linearized plasmids were transfected into 293A cells (Life Technologies) by using Fugene 6 transfection reagent (Roche Diagnostics, Basel, Switzerland). The viruses were propagated in 293A cells and purified using CsCl_2_ banding followed by dialysis against 10 mM Tris-HCl, pH 8.0 with 2 mM MgCl. The infectious titers were determined using the Adeno-X Rapid Titer kit (Takara, Shiga, Japan). The successful adenoviral gene transfer is shown in Figure S1A, in which 5×10^9^ infectious units of the viruses were injected into wild-type mice, and immunohistochemistry using the target antibodies was performed 3 days after the infection. 39.6±3.9%, 26.4±5.8%, 46.5±3.2%, 57.3±5.8%, of liver cells reacted to PDX1, NEUROD, MAFA, and MAFB antibodies, respectively (n = 3) (Figure S1A).

### Bioluminescence imaging

Adenoviral constructs (5×10^9^ IFU) expressing GFP (Ad-GFP), PDX1, NEUROD, and MAFA (Ad-PDA) and PDX1, NEUROD, and MAFB (Ad-PDB) were intravenously injected into the MIP-Luc-VU mice. Serial BLI was performed before and after infection on the designated days. The day of injection was determined as day 0. To detect the bioluminescence signals emitted from the free-fed mice, D-luciferin (5 mg/kg body weight; Promega, Madison, WI, USA) was injected intraperitoneally, and bioluminescence images were taken using an IVIS spectrum 5 minutes later (Caliper Life Sciences, Hopkinton, MA, USA). Bioluminescence images were captured with an integration time of 1 minute, and isometric regions of interest (ROIs) were drawn over the location corresponding to the liver for the quantification using Living Image software (Xenogen Corporation, Alameda, CA, USA).

### Quantitative RT-PCR

Total RNA was extracted from the liver using the NucleoSpin RNA II kit (Machery-Nagel, Düren, Germany), and 1 µg was used to prepare cDNA primed with random hexamers and reverse-transcribed with the QuantiTect Reverse Transcription kit (Qiagen, Hilden, Germany) according to the manufacturer's protocol. Gene expression levels were compared by real-time RT-PCR using the Thermal Cycler Dice Real Time system (Takara) with a SYBR Green PCR master mix (Takara). Values were normalized to expression levels of *Hprt* and shown in relative amount to the PDA group at day 3. All primer sequences are listed in Table 1 [15].

**Table 1:** Primer sequences used in the study.

Gene symbol	Forward primer sequences	Reverse primer sequences
*Ins1*	5′-CCAGCTATAATCAGAGACCA-3′	5′-GGGCCTTAGTTGCAGTAGTT-3′
*Ins2*	5′-AGGAAGCCTATCTTCCAGGT-3′	5′-ATTCATTGCAGAGGGGTAGG-3′
*Slc2a2*	5′-TGGGATGAAGAGGAGACTGAA-3′	5′-CATCCGTGAAGAGCTGGATCA-3′
*Pcsk2*	5′-AATGACCCCTAACCCATACCC-3′	5′-GAGGAGGGTTCGATGATGTC-3′
*islet Gck*	5′-TGGATGACAGAGCCAGGATGG-3′	5′-ACTTCTGAGCCTTCTGGGGTG-3′
*Sur1*	5′-CTGGTCCTCAGCAGCACAT-3′	5′-GGAACTCTTGGGACGAGACA-3′
*Kcnj11 (Kir6.2)*	5′-GTAGGGGACCTCGGAAAGAG-3′	5′-TGGAGTCGATGACGTGGTAG-3′
*Sytl4*	5′-CCAGCACACAAAGGCGAGT-3′	5′-GAAGGAGGTATCCCTTGACAAAG-3′
*Hprt*	5′-GGCTTCCTCCTCAGACCGCTTT-3′	5′-AGGCTTTGTATTTGGCTTTTCC-3′

### Measurement of insulin content

Hepatic insulin after adenovirus infection was extracted by the acid-ethanol method as described previously, and the content measured with an insulin enzyme-linked immunosorbent assay kit (Morinaga Bioscience, Yokohama, Japan) [16]. The insulin content was normalized to the total protein concentration measured by a Coomassie protein assay reagent (Thermo Scientific, Hudson, NH, USA).

### Diabetes induction

Wild-type mice were rendered diabetic at 6 weeks of age by IP injection of streptozotocin (STZ, Sigma) at a dose of 200 mg/kg body weight in 0.1 M citrate buffer (pH 4.5). The serum glucose concentrations of the mice after they had been subjected to a 12-hour overnight fast were measured using a Dry-chem 3500 automated analyzer for routine laboratory tests (Fujifilm, Tokyo, Japan). The serum insulin levels were measured with an ultrasensitive insulin enzyme-linked immunosorbent assay kit (Morinaga).

### Immunostaining

The MIP-Luc-VU mice were euthanized at 8 weeks of age, and the livers removed. Tissues were fixed in 10% formalin and embedded in paraffin. The tissue sections were incubated with anti-insulin antibody (Abcam, Cambridge, UK) for 8 hours at 4°C. The antigens were visualized using appropriate secondary antibody conjugated with Alexa 596 with nuclear staining using 4′,6-diamidino-2-phenylindole (DAPI) (Invitrogen). Images of the sections were scanned and analyzed using a Biorevo BZ-9000 microscope (Keyence, Osaka, Japan) and BZ-II Analyzer software (Keyence).

### *In situ* liver perfusion

The experiment was performed according to a previous report by Yechoor et al [17]. Briefly, anesthetized mice were cannulated via the portal vein for infusion and via the inferior vena cava (IVC) through the right atrium for collection of the effusate. After the initial washout, a peristaltic pump was used to perfuse the isolated liver with KRB buffer (119 mM NaCl, 4.7 mM KCl, 25 mM NaHCO_3_, 2.5 mM CaCl_2_, 1.2 mM MgSO_4_, 1.2 mM KH_2_PO_4_, and 0.25% BSA) containing different concentrations of glucose. Finally, after another washout, glibenclamide (10 nM; Sigma) and KCl (25 mM) were added. The insulin concentration of the effusate was measured 10 minutes after each perfusion of glucose.

### Statistical analysis

Data were expressed as the means ± standard errors of the means and compared using an unpaired *t* test, unless otherwise stated. Probability values of less than 0.05 were considered significant.

## Results

**Figure 1:**
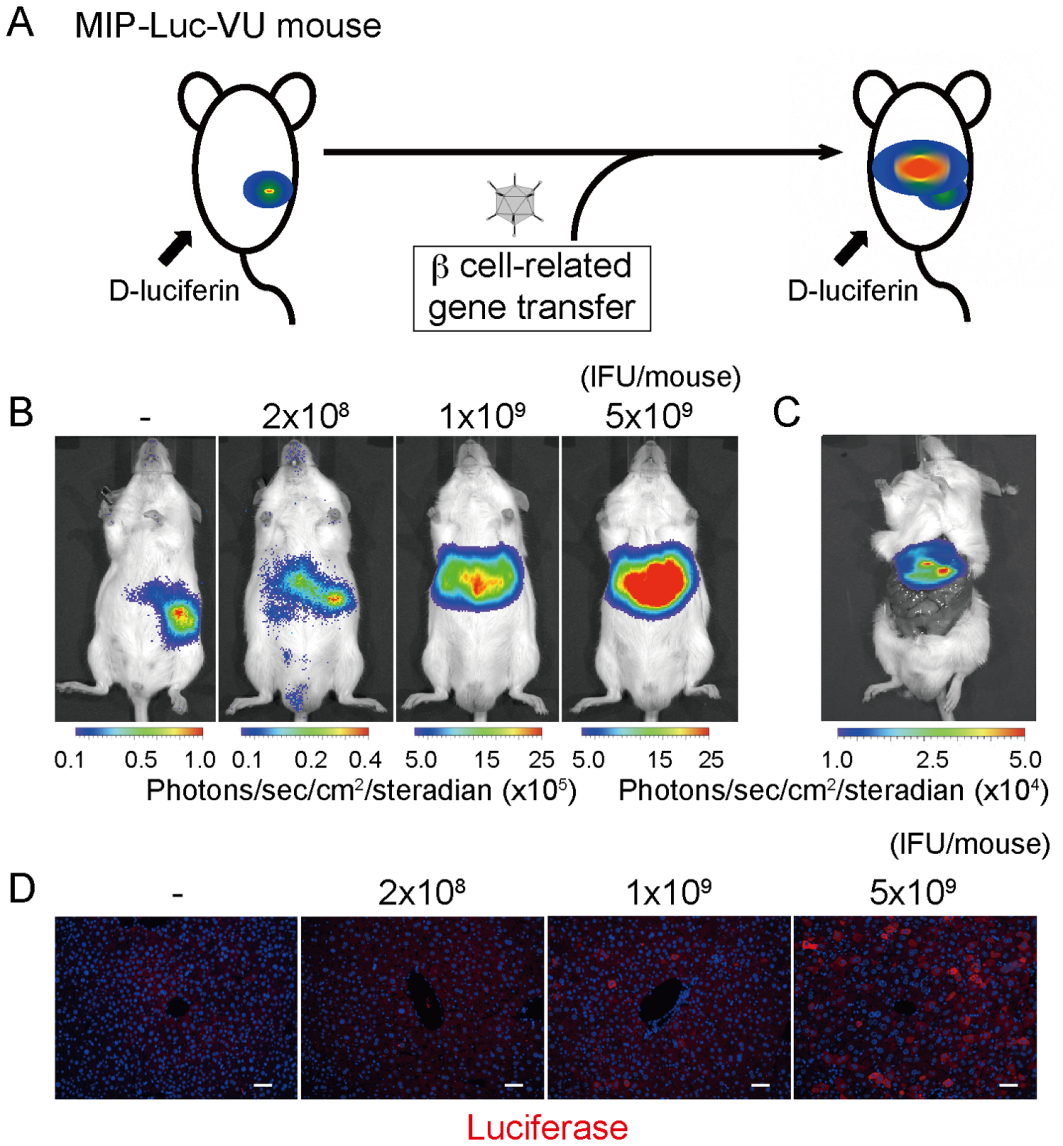
Bioluminescence emission from the hepatic region of MIP-Luc-VU mice after β cell-related gene transfer. (A) Diagrammatic representation of bioluminescence monitoring of insulin transcriptional activity. (B) Representative example of dose-dependent bioluminescence emission from the hepatic region. (C) Bioluminescence images of the abdominal section of MIP-Luc-VU mice 3 days after infection. (D) Tissue sections of MIP-Luc-VU liver stained with anti-luciferase antibody with 4′,6-diamidino-2-phenylindole (DAPI) 3 days after gene transfer. Scale bar indicates 100 µm.

To screen the transcriptional activity of the intrahepatic insulin gene in a noninvasive manner, we monitored the bioluminescence emissions from MIP-Luc-VU mice after adenovirus-mediated gene transfer (Figure 1A). We transferred a 3-gene combination of *Pdx1*, *Neurod*, and *Mafa* into the MIP-Luc-VU mice with different infectious titers to detect the bioluminescence signal 3 days after infection, according to a previous study (Figure 1B) [18]. After luciferin injection (5 mg/kg body weight, IP) into the gene-transferred mouse, within minutes the emission from the liver rose quickly and then gradually attenuated (Figure S1B). The 3-gene transfer resulted in a dose-dependent bioluminescence emission in the hepatic region, which was identified as originating from the liver by imaging of the extracted tissues and by immunohistochemistry using anti-luciferase antibody (Figure 1B, C, D). Most hepatic cells exhibit reporter expression, whereas immunohistochemistry using anti-insulin antibody showed very few luciferase-positive cells expressing insulin protein, indicating the existence of some regulatory mechanisms of the transcription and the protein levels of insulin, such as mRNA and protein degradation and leakage of produced insulin into the blood circulation (Figure S1C).

**Figure 2:**
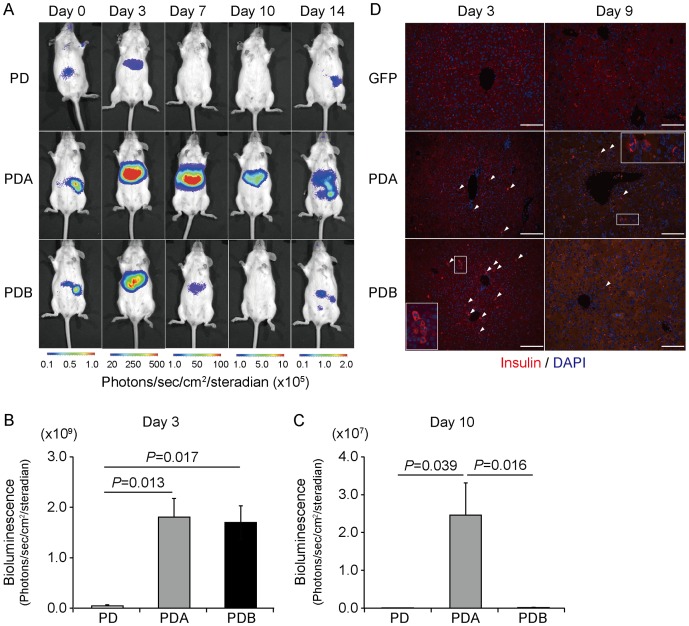
Comparison of the transcription of insulin in the liver by use of bioluminescence imaging. (A) Representative bioluminescence imaging of MIP-Luc-VU mice after *Pdx1*/*Neurod* (PD), *Pdx1*/*Neurod*/*Mafa* (PDA), and *Pdx1*/*Neurod*/*Mafb* (PDB) gene transfer. (B, C) Quantification of signal intensity after PD (n = 6), PDA (n = 7), and PDB (n = 7) gene transfer at days 3 (B) and 10 (C). (D) Tissue sections of wild-type mouse liver stained with anti-insulin antibody (red) and 4′6-diamidino-2-phenylindole (DAPI) (blue) after GFP-, PDA-, and PDB-gene transfer. Arrowheads indicate insulin-positive cells. Scale bars indicate 100 µm.

**Figure 3:**
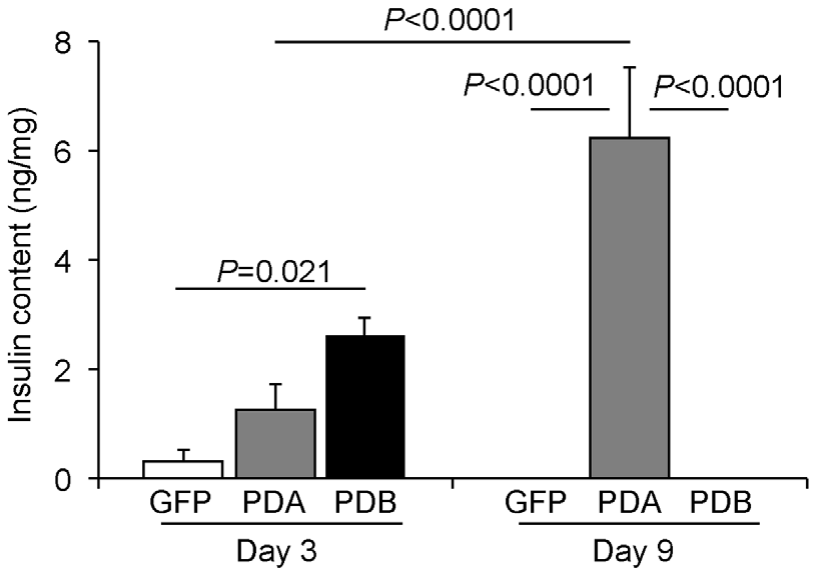
Insulin content in mouse liver after β-like cell-related gene transfer. Insulin content of wild-type mouse liver at days 3 and 9 after GFP (n = 7), *Pdx1*/*Neurod*/*Mafa* (PDA, n = 8), and *Pdx1*/*Neurod*/*Mafb* (PDB, n = 5) gene transfer. Data were expressed as the means ± standard errors of the means and analyzed by one-way ANOVA followed by the Tukey-Kramer honestly significant difference (HSD) test.

To compare the effects of *Mafa* and *Mafb* on the transcriptional activity of the insulin gene through measurement of the bioluminescence emission, we examined 3 different gene combinations – *Pdx1*/*Neurod* (PD); *Pdx1*, *Neurod*, and *Mafa* (PDA); and *Pdx1*, *Neurod*, and *Mafb* (PDB) – in MIP-Luc-VU mice (Figure 2A). The bioluminescence signals induced by the 3 different combinations peaked at day 3 and gradually disappeared over 14 days in the same way. The PDA (1.81±0.34×10^9^/photons/sec/cm^2^/steradian; n = 7) and PDB (1.70±0.33×10^9^/photons/sec/cm^2^/steradian; n = 8) transductions induced comparable but significantly higher signal intensities than the peak emission of PD (4.49±2.6×10^7^/photons/sec/cm^2^/steradian; n = 4) at day 3 (Figure 2B). In contrast, only the PDA-transferred mice continued to emit substantial bioluminescence, even at day 10, suggesting that *Mafa* is capable of inducing a peak emission similar to but a more sustainable insulin gene activity than that of *Mafb* (PD: 1.28±0.1×10^5^/photons/sec/cm^2^/steradian; n = 4, PDA: 2.46±0.8×10^7^/photons/sec/cm^2^/steradian; n = 7, PDB: 1.96±0.4×10^5^/photons/sec/cm^2^/steradian; n = 8) (Figure 2C). Furthermore, immunohistochemistry using anti-insulin antibody revealed that some PDA- and PDB-transferred liver cells reacted to the antibody at days 3 and 9 after infection (Figure 2D). To confirm the presence of insulin-positive cells in PDB-transferred liver, DAB (3, 3′-diaminobenzidine) visualization is also shown in Figure S2A. We next examined the insulin content of the gene-transferred livers at days 3 and 9 after infection (Figure 3). Consistent with the bioluminescence results, we found no difference among the groups at day 3 (GFP: 0.31±0.2 ng/mg; n = 4; PDA: 1.26±0.5 ng/mg; n = 7; PDB: 2.6±0.3 ng/mg; n = 5), but the PDA-transferred liver at day 9 (6.23±1.3 ng/mg, n = 4) contained fairly abundant amounts of insulin protein when compared with the other groups. Interestingly, the insulin content of the PDA-transferred liver at day 9 was increased 5-fold over that of day 3, suggesting that newly synthesized insulin protein is gradually stored in the liver.

**Figure 4:**
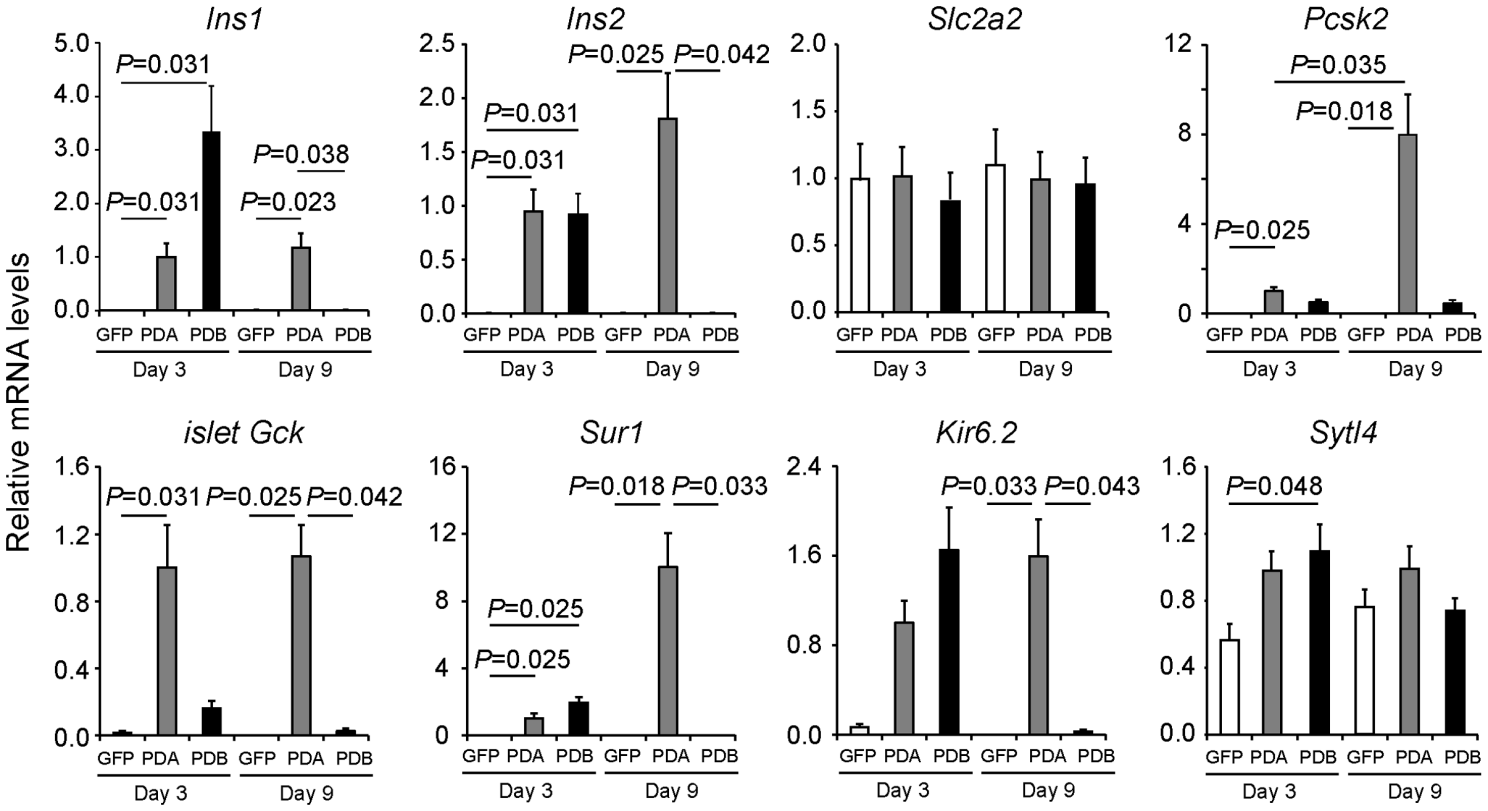
Expression of β cell-related genes in mouse liver after *Pdx1/Neurod* in combination with *Mafa* (PDA) or *Mafb* (PDB) gene transfer. Wild-type mouse liver treated with Ad-PDA (PDA, n = 7) displayed a significant increase in β cell-related mRNAs at day 9 (*Ins1*, *Ins2*, *PC2*, *i-GK*, *Sur1*, *Kir6.2*) on Q-PCR analysis. In contrast, the Ad-PDB treated group of mice (PDB, n = 7) displayed a significant increase in *Ins1*, *Ins2*, *Sur1*, and *Kir6.2* only at day 3. Data were normalized to *Hprt* mRNA abundance and shown as relative expression levels to that of the Ad-PDA-treated group at day 3. Data were expressed as the means ± standard errors of the means and analyzed using the Steel-Dwass multiple comparison test.

To examine the gene expression profiles related to β-cell function, we performed quantitative RT-PCR analysis using RNAs recovered from whole liver cells (GFP, n = 6) (Figure 4). The expressions of *Pdx*1, *Neurod*, *Mafa*, and *Mafb* are shown in Figure S2B. Both Ad-PDA (n = 7) and Ad-PDB (n = 7) treatment induced comparable expression levels of β cell-related genes, including *Slc2a2* and *Pcsk2*, 3 days after infection, but only in the PDA-transfer group were the expressions sustained for longer than 1 week. In contrast to this significant β cell-related gene induction, immunohistochemistry showed that other hormones including glucagon, pancreatic polypeptide (PP), and somatostatin were not induced by the Ad-PDA and Ad-PDB treatments (data not shown). Furthermore, other β cell-specific transcription factors, such as *Nkx2.2*, *Nkx6.2*, and *Pax4* that are detected in normal β-cell development, were neither induced nor increased by PDA-gene transfer, suggesting that transient β-like cell induction does not occur in the similar molecular cascade to that of the regular developmental process of β cells.

**Figure 5:**
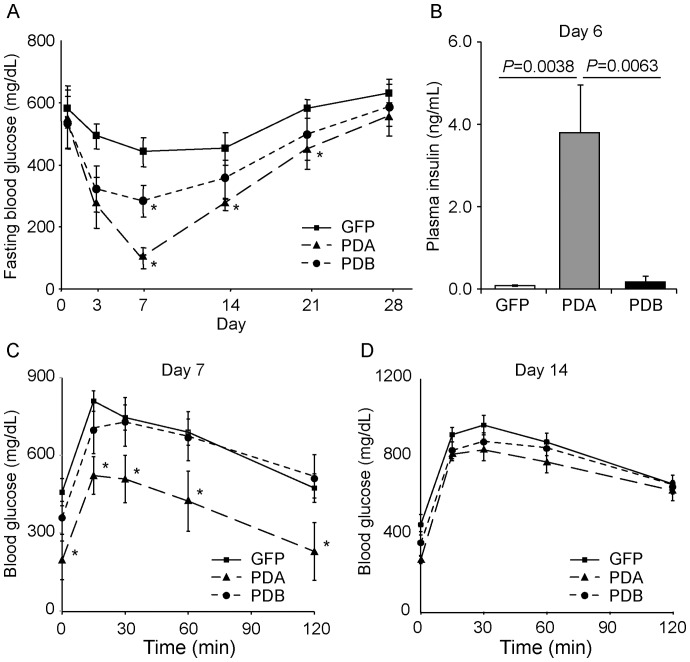
*Pdx1*, *Neurod*, and *Mafa* gene transfer in the liver significantly improved blood glucose levels in a streptozotocin-induced diabetic model in comparison with *Pdx1*, *Neurod*, and *Mafb* gene transfer. (A) ICR mice were rendered diabetic by intraperitoneal injection of streptozotocin (STZ; 200 mg/kg) followed by treatment with Ad-GFP, Ad-PDA, or Ad-PDB (2.5×10^9^ IFU). Fasting blood glucose levels were measured 0 to 28 days after the treatment. Data were expressed as the means ± standard errors of the means and analyzed using the Steel-Dwass multiple comparison test. (B) Plasma insulin levels in the fasting state 6 days after the treatment. Data were expressed as the means ± standard errors of the means, and *P* values calculated using the Tukey-Kramer honestly significant difference (HSD) test. (C–D) Intraperitoneal glucose tolerance tests 7 (C) and 14 (D) days after the treatment. After an overnight fast, the STZ-induced diabetic mice were injected intraperitoneally with 2.0 g/kg body weight of glucose. Blood samples collected at the designated times after glucose challenge were used to measure blood glucose levels. Data were expressed as the means ± standard errors of the means, and *P* values calculated using the Tukey-Kramer HSD test. *P<0.05.

Next, to examine whether hepatic insulin production induced by PDA can ameliorate blood glucose levels in the diabetic model, we injected 200 mg/kg STZ into ICR mice, followed by control Ad-GFP (n = 8) or Ad-PDA (n = 5) treatment on day 7 after diabetes induction. As shown in Figure 5A, fasting blood glucose levels were recovered by Ad-PDA treatment for up to 21 days, which was more pronounced and persistent than the effects of Ad-PDB treatment (n = 7) (Figure 5A). Consistent with this result, serum insulin levels after 12 hours of fasting in the mice treated with Ad-PDA (3.87±1.2 ng/ml, n = 4) were significantly higher than those in the mice treated with Ad-GFP (0.088±0.02 ng/ml, n = 5) or Ad-PDB (0.18±0.07 ng/ml, n = 4) (Figure 5B). Furthermore, we also performed an intraperitoneal glucose tolerance test 7 and 14 days after the adenovirus injection. As shown in Figure 5C (7 days) and 5D (14 days), the Ad-PDA-treated mice showed lower glucose levels at each time point after the glucose stimulation; however, the reduction in blood glucose levels in the Ad-PDA-treated mice (n = 4) was very consistent at all time points – about 150 mg/dL (day 7) and 50 mg/dL (day 14) – when compared with those in the Ad-GFP-treated mice (n = 6). These results raise the possibility that Ad-PDA treatment could not give rise to mature insulin-producing cells capable of secreting insulin in response to glucose levels despite the significant improvement in fasting blood glucose levels in the STZ diabetic model.

**Figure 6:**
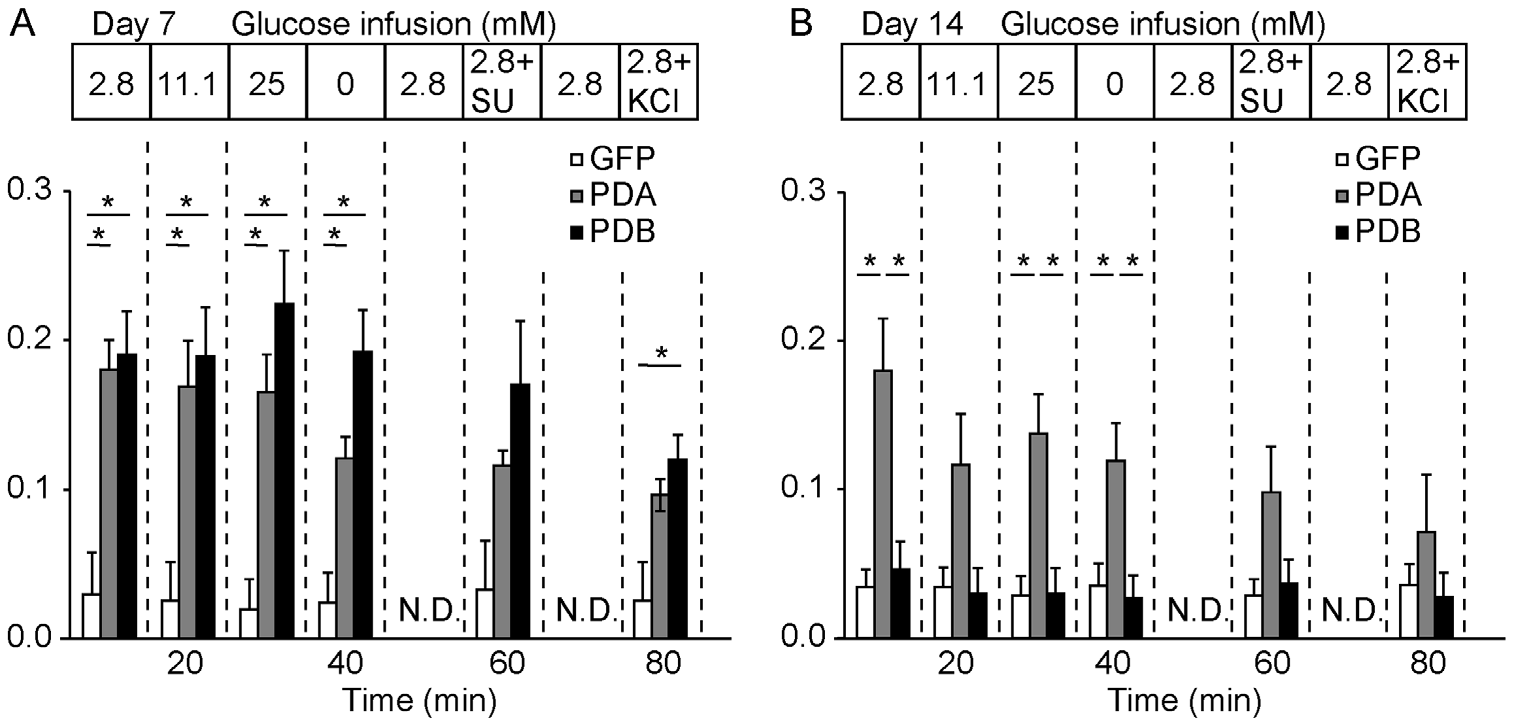
Glucose-independent insulin release from the mouse liver after β-like cell-related gene transfer. (A–B) Glucose-stimulated insulin release from the livers of Ad-GFP- (n = 6), Ad-PDA- (n = 6), and Ad-PDB- (n = 7) treated mice 7 days (A) and 14 days (B) after treatment by *in situ* liver perfusion. Data were expressed as the means ± standard errors of the means, and *P* values calculated using the Tukey-Kramer honestly significant difference (HSD) test. *P<0.05. N.D. indicates no data.

Finally, to examine directly whether PDA-transferred liver cells are capable of secreting insulin in respond to glucose stimulation, we performed an *in situ* liver perfusion experiment (Figure 6A, B). Solutions containing different glucose concentrations were infused via the portal vein, and the liver effusion was collected for the measurement of insulin and glucose concentrations. In contrast to the Ad-GFP treatment, the Ad-PDA and Ad-PDB treatments led to high insulin secretion at all time points; however, stepwise increase in insulin secretion did not occur during glucose stimulation in the infusate from 2.8 to 11.1 and 25 mM. Consistent with the result of the intraperitoneal glucose tolerance test, this result indicates that liver cells after PDA-gene transfer secrete insulin independent of glucose concentration, similar to basal insulin secretion.

## Discussion

In this study, we used β-like cell induction in the mouse liver to examine the functional differences between 2 large Maf transcription factor genes that are expressed in the pancreatic islets. Although no reports have been published on the successful generation of β-like cells by overexpression of gene combination including *Mafb* by other types of cells, we transiently but successfully induced insulin-producing cells by overexpression of *Mafb* in combination with *Pdx1* and *Neurod* [19]. However, hepatic insulin-producing cells induced by Ad-PDB treatment demonstrated defective expressions of *Pcsk2* and *islet Gck* (Figure 4) and insulin release in a glucose-independent manner (Figures 5, 6), suggesting that *Mafb* is capable of stimulating β-cell conversion to the immature and transitional states [20]. Meanwhile, the possibility remained that PDB and PDA transfer induced a few mature β cells, the phenotype of which is masked by the majority of immature β cells. Notably, MAFB^+^ insulin^+^ cells are generated during the normal β-cell differentiation process before the emergence of MAFA^+^ insulin^+^ cells, implying that the transition from *Mafb* to *Mafa* is critical to β-cell maturation [5], [7]. Meanwhile, β cells acquire the capability of glucose-stimulated insulin secretion during postnatal days 0 to 10, when downregulation of *Mafb* expression takes place in β cells [21], [22]. Therefore, alteration of gene expression profiles after birth might also be considered for deeper understanding of the molecular mechanism of β-cell maturation [23].

Large Maf transcription family proteins show relatively high levels of amino acid similarity in each domain. In mice, MAFB shares 57%, 86%, and 72% amino acid sequence identities with the individual acidic, basic, and leucine zipper moieties of MAFA, respectively [24]. Although we have not determined which amino acids or regions in MAFA are dominant for the generation of β-like cells from liver cells, the C-terminal DNA-binding region of MAFA has been shown to be critical for insulin induction [19]. In addition, the difference in activity between MAFA and MAFB might also be related to posttranslational modifications, including phosphorylation, ubiquitination, and sumoylation [25]–[30]. Recent studies using gene knockout mice revealed that *Mafa* and *Mafb* regulate different genes critical for glucose-stimulated insulin secretion and β-cell development, respectively, supporting our results indicating the different conversion capabilities of these 2 genes [31].

We demonstrated that bioluminescence monitoring using an apparatus equipped with a sensitive CCD camera capable of detecting slight bioluminescence from reporter mice could be useful for noninvasive monitoring of intrahepatic insulin gene activity *in vivo*. Indeed, it has been demonstrated that hepatocytes or hepatocyte precursors could be converted to insulin-producing cells using different gene combinations [17], [18], [32]–[34]. However, a comprehensive method for comparing these protocols and determining the optimal conditions for β-cell generation has not been established [14]. Our simple and repetitive monitoring described here provides readily quantifiable data to examine the efficiency of β-cell induction under different protocols.

In this study, we transduced the same PDA gene combination as that of a previous report by Kaneto et al [18]. In contrast to ours, their results demonstrated fairly good insulin secretion in response to glucose stimulation. This discrepancy is probably due to the different mouse strains and PDX1 used. They injected adenovirus expressing a constitutively active form of PDX1, PDX1-VP16 fusion protein, into C57BL/6 mice, whereas we used wild-type PDX1 and ICR mice. Especially, the PDX1-VP16 form, of which VP16 is the activation domain from the herpes simplex virus, has been demonstrated to show potent transdifferentiation activity when compared with wild-type PDX1 [35], [36].

In summary, we have demonstrated that bioluminescence imaging in MIP-Luc-VU mice provides a noninvasive means of monitoring insulin transcriptional activity and the presence of β-like cells in the liver, and *Mafa* has a markedly sustainable role in β-like cell induction in comparison with *Mafb*.

## Supporting Information

Figure S1 (A) Immunohistochemistry of wild-type mouse liver infected with the indicated viruses 3 days after induction. Liver tissues were fixed in 4% paraformaldehyde overnight and embedded in OCT compound (Sakura, Tokyo, Japan). The tissue sections were incubated with anti-PDX1 (Abcam, ab47267), NEUROD (Santa Cruz, sc-1084), MAFA (Bethyl, BL1069) and MAFB (Bethyl, IHC-00351) antibodies and visualized using appropriate secondary antibodies conjugated with Alexa 596 with nuclear staining using 4′,6-diamidino-2-phenylindole (DAPI) (Invitrogen). Lower panels indicate negative control sections. Scale bars indicate 50 µm. (B) Bioluminescence emission from the liver of PDA-transferred MIP-Luc-VU mice following intraperitoneal injection of luciferin. (C) Tissue sections of MIP-Luc-VU liver stained with anti-luciferase (Promega, G475A) and anti-insulin (Abcam, ab7842) antibodies with 4′,6-diamidino-2-phenylindole (DAPI) 3 days after PDA gene transfer. Scale bar indicates 100 µm.
(TIF)

Figure S2 (A) Immunohistochemistry for anti-insulin antibody in the livers treated with Ad-GFP (Left), Ad-PDB (Center) and islets treated with Ad-PDB (Right). Scale bars indicate 50 µm. (B) Expression of genes delivered in mouse liver treated with Ad-GFP, Ad-PDA, and Ad-PDB. Primer sequences: 5′-TTCCCGAATGGAACCGAGC-3′ and 5′-GTAGGCAGTACGGGTCCTCT-3′ for *Pdx1*; 5′-ACAGACGCTCTGCAAAGGTTT-3′ and 5′-GGACTGGTAGGAGTAGGGATG-3′ for *Neurod*; 5′-CACTGGCCATCGAGTAGTCA-3′ and 5′-CTTCACCTCGAACTTCATCAGGTC-3′ for *Mafa*; 5′-TGAGCATGGGGCAAGAGCTG-3′ and 5′-CCATCCAGTACAGGTCCTCG-3′ for *Mafb*; and 5′-GCTATGTGAGCACTCCACAG-3′ and 5′-CCATCCAGTACAGGTCCTCG-3′ for exogenous specific *Mafb*.
(TIF)
