# Supplementary figures and images for: Generation of Insulin-Producing Cells from the Mouse Liver Using β Cell-Related Gene Transfer Including Mafa and Mafb
Source: PLoS One. 2014 Nov 14;9(11):e113022. doi: 10.1371/journal.pone.0113022 (PMC4232560; doi:10.1371/journal.pone.0113022)

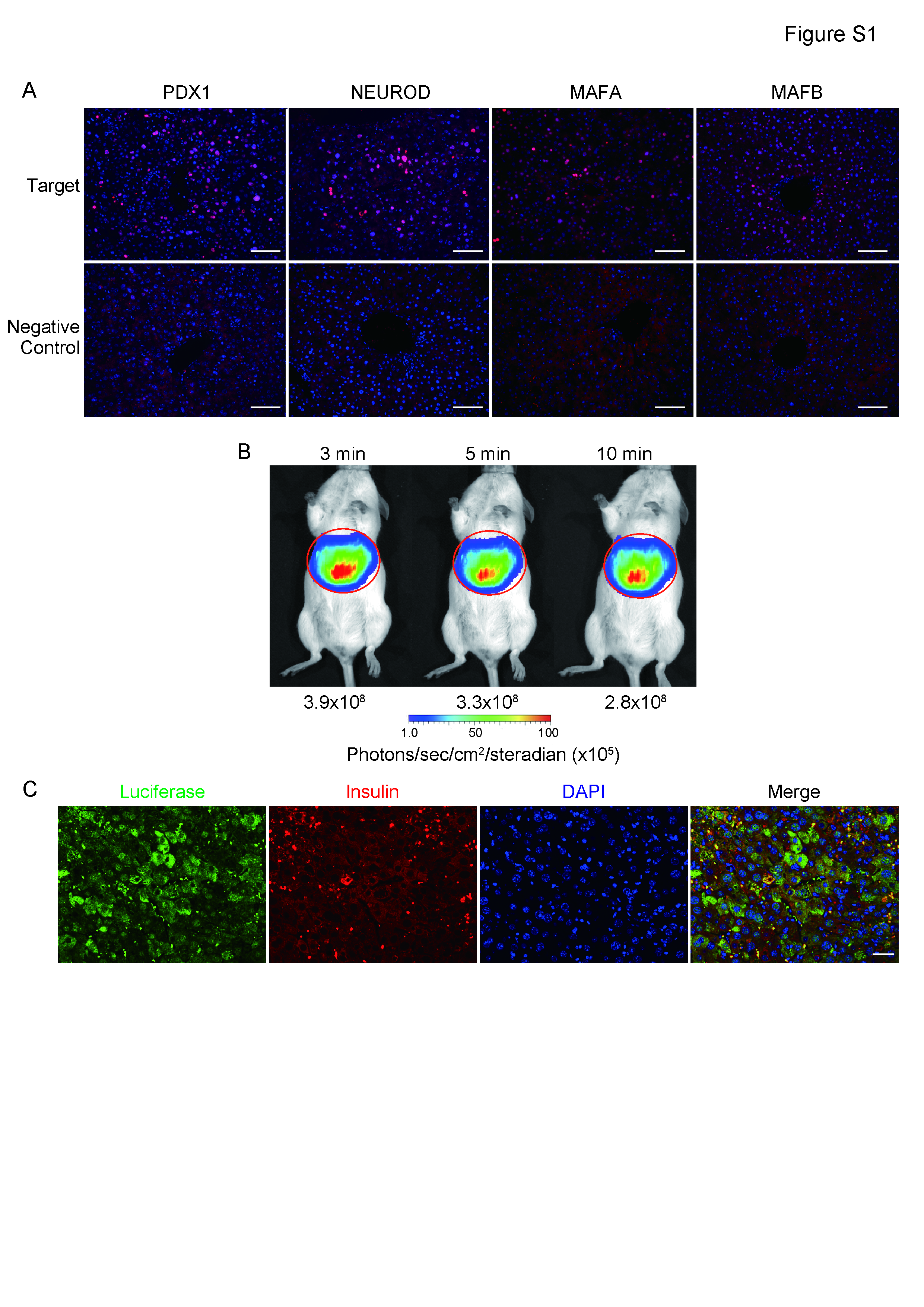

Supplement: Figure S1 — (A) Immunohistochemistry of wild-type mouse liver infected with the indicated viruses 3 days after induction. Liver tissues were fixed in 4% paraformaldehyde overnight and embedded in OCT compound (Sakura, Tokyo, Japan). The tissue sections were incubated with anti-PDX1 (Abcam, ab47267), NEUROD (Santa Cruz, sc-1084), MAFA (Bethyl, BL1069) and MAFB (Bethyl, IHC-00351) antibodies and visualized using appropriate secondary antibodies conjugated with Alexa 596 with nuclear staining using 4′,6-diamidino-2-phenylindole (DAPI) (Invitrogen). Lower panels indicate negative control sections. Scale bars indicate 50 µm. (B) Bioluminescence emission from the liver of PDA-transferred MIP-Luc-VU mice following intraperitoneal injection of luciferin. (C) Tissue sections of MIP-Luc-VU liver stained with anti-luciferase (Promega, G475A) and anti-insulin (Abcam, ab7842) antibodies with 4′,6-diamidino-2-phenylindole (DAPI) 3 days after PDA gene transfer. Scale bar indicates 100 µm. (TIF) [file pone.0113022.s001.tif]

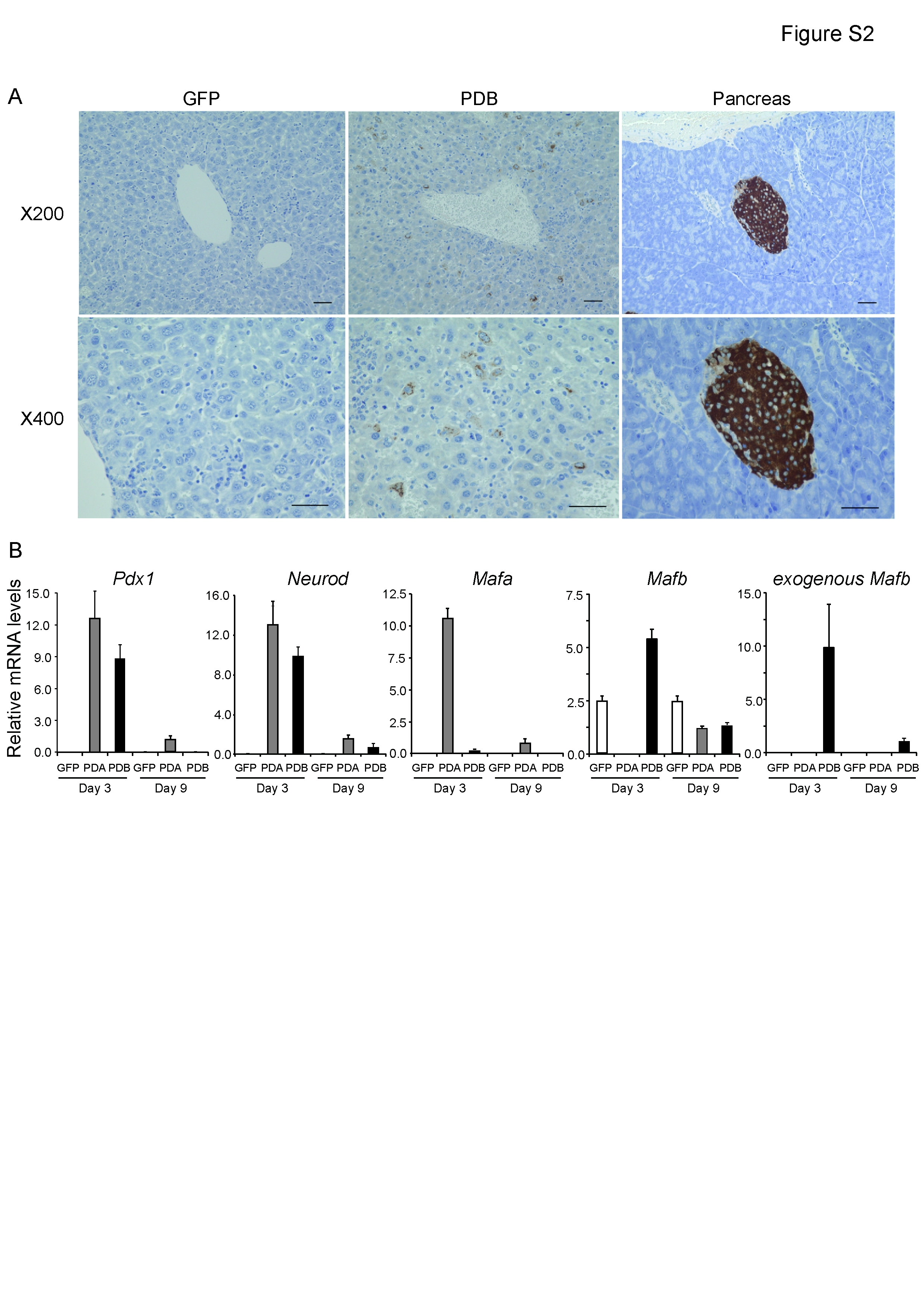

Supplement: Figure S2 — (A) Immunohistochemistry for anti-insulin antibody in the livers treated with Ad-GFP (Left), Ad-PDB (Center) and islets treated with Ad-PDB (Right). Scale bars indicate 50 µm. (B) Expression of genes delivered in mouse liver treated with Ad-GFP, Ad-PDA, and Ad-PDB. Primer sequences: 5′-TTCCCGAATGGAACCGAGC-3′ and 5′-GTAGGCAGTACGGGTCCTCT-3′ for Pdx1; 5′-ACAGACGCTCTGCAAAGGTTT-3′ and 5′-GGACTGGTAGGAGTAGGGATG-3′ for Neurod; 5′-CACTGGCCATCGAGTAGTCA-3′ and 5′-CTTCACCTCGAACTTCATCAGGTC-3′ for Mafa; 5′-TGAGCATGGGGCAAGAGCTG-3′ and 5′-CCATCCAGTACAGGTCCTCG-3′ for Mafb; and 5′-GCTATGTGAGCACTCCACAG-3′ and 5′-CCATCCAGTACAGGTCCTCG-3′ for exogenous specific Mafb. (TIF) [file pone.0113022.s002.tif]
